# Adolescent alcohol and cannabis use as risk factors for head trauma in the Northern Finland Birth Cohort study 1986

**DOI:** 10.1093/eurpub/ckad151

**Published:** 2023-08-23

**Authors:** Maarit K Koivisto, Jussi Puljula, Jonna M Levola, Antti Mustonen, Jouko Miettunen, Anni-Emilia Alakokkare, Solja Niemelä

**Affiliations:** Department of Psychiatry, University of Turku, Turku, Finland; Emergency Services, TYKS Acute, Turku University Hospital, Turku, Finland; Department of Neurology, Lapland Central Hospital, Rovaniemi, Finland; Department of Psychiatry, University of Helsinki and Helsinki University Hospital, Helsinki, Finland; Faculty of Medicine and Health Technology, University Consortium of Seinäjoki, Tampere University, Tampere, Finland; Center for Life Course Health Research, University of Oulu, Oulu, Finland; Department of Psychiatry, Seinäjoki Central Hospital, Seinäjoki, Finland; Center for Life Course Health Research, University of Oulu, Oulu, Finland; Medical Research Center Oulu, Oulu University Hospital and University of Oulu, Oulu, Finland; Department of Psychiatry, University of Turku, Turku, Finland; Center for Life Course Health Research, University of Oulu, Oulu, Finland; Department of Psychiatry, University of Turku, Turku, Finland; Addiction Psychiatry Unit, Department of Psychiatry, Hospital District of South-West, Turku, Finland

## Abstract

**Background:**

The aim of this study was to assess the associations between cannabis use and frequency of alcohol intoxication in adolescence with the risk of traumatic brain injury and craniofacial fractures in early adulthood. Hypothesis was that using alcohol and cannabis in adolescence could increase the risk for head traumas.

**Methods:**

Data from the Northern Finland Birth Cohort 1986 (*n* = 9432 individuals) were used to investigate the prospective association between the self-reported frequency of alcohol intoxication (*n* = 6472) and cannabis use (*n* = 6586) in mid-adolescence and register-based, head trauma diagnoses by ages 32–33 years. To test the robustness of these associations, the statistical models were adjusted for a range of other confounders such as illicit drug use, previous head trauma and self-reported mental health problems.

**Results:**

In multivariate analyses, cannabis use was statistically significantly associated with a greater risk of traumatic brain injury among females [hazard ratio (HR) 1.9, 95% confidence interval (CI) 1.1–3.2, *P* = 0.024). Frequent alcohol intoxication was a statistically significant independent risk factor for both traumatic brain injury (HR 2.6, 95% CI 1.7–3.9, *P* < 0.001) and craniofacial fractures (HR 2.7, 95% CI 1.6–4.8, *P* < 0.001) among males.

**Conclusions:**

Cannabis use in adolescence appears to associate independently with elevated risk for traumatic brain injury among females, and frequent alcohol intoxication in adolescence seems to associate with elevated risk of both traumatic brain injury and craniofacial fractures among males.

## Introduction

Traumatic brain injuries (TBIs) are a considerable public health issue and societal burden worldwide.^1–3^ TBIs are a major cause of mortality and disability^2^ and cause over 2 million hospital admissions and 80 000 deaths annually in Europe alone.^3^ Previous studies report that alcohol use is a contributor in 30–50% of TBI cases^1^^,^^3^^,^^4^ and that alcohol use disorder (AUD) independently associates with TBI.^5^ Among adolescents and young adults, males have a higher risk for TBIs.^2^^,^^3^^,^^5^^,^^6^ Reported associations of TBIs and substance use behaviors might be reciprocal, because TBIs have been also associated with subsequent mental health problems in previous prospective studies. Evidence of TBIs as a risk factor for substance use disorders (SUD) in later life has remained inconclusive.^7–10^

Cannabis use has become more common during the past decade, especially among adolescents and young adults.^11^^,^^12^ Adolescent cannabis use has been associated with not only impaired mental health and cognitive skills but also higher risk of traffic accidents.^13^^,^^14^ The use of substances other than alcohol is common before TBI,^15^ but there is little knowledge about the relationship between the use of these substances (especially cannabis use) and the risk of TBI in later life.

Adolescent alcohol use has been shown to be associated with mental health problems and psychiatric morbidity in young adulthood.^16–18^ Furthermore, frequent alcohol intoxication in adolescence is associated with a higher prevalence of SUD in later life and with premature mortality.^19–21^ To our knowledge, there is only one birth cohort study that has examined adolescent drinking habits as risk factors for TBI in early adulthood.^22^ That study utilized the Northern Finland Birth Cohort (NFBC) 1966 and included 10 424 adolescents with a 35-year follow-up. That study reported an association between frequent (at least monthly) adolescent alcohol drinking and elevated risk of TBI in later life [hazard ratio (HR) 2.21, 95% confidence interval (CI) 1.14–4.29]. However, that study evaluated lifetime alcohol use and alcohol intoxication; thus, we are lacking data over a specific time period. Furthermore, that study did not account for a range of other relevant confounders such as previous head trauma.^22^

In the present study, we used data from the NFBC 1986^23^ to investigate the prospective association between the self-reported frequency of alcohol intoxication and cannabis use in mid-adolescence and register-based, head trauma diagnoses by ages 32–33 years. Additionally, we aimed to test the robustness of these associations by adjusting for a range of other confounders such as illicit drug use, previous head trauma and self-reported mental health problems. We hypothesized that using alcohol and cannabis in adolescence could increase the risk for TBI.

## Methods

### Participants

NFBC1986 is an ongoing follow-up study of 99% of all births, including all live-born children (*n* = 9432) with an expected birth between 1 July 1985 and 30 June 1986 from the two northernmost provinces in Finland (University of Oulu^23^). The data collection commenced in two phases when the participants were aged 15–16 years: first by a postal questionnaire study for adolescents and parents (conducted between April 2001 and February 2002), followed by a clinical flied study (conducted between August 2001 and June 2002) in which the participants completed a supplementary questionnaire that included questions on their alcohol and drug use (University of Oulu^23^). Participants were included in the study if they signed the informed consent form.

**Figure 1:**
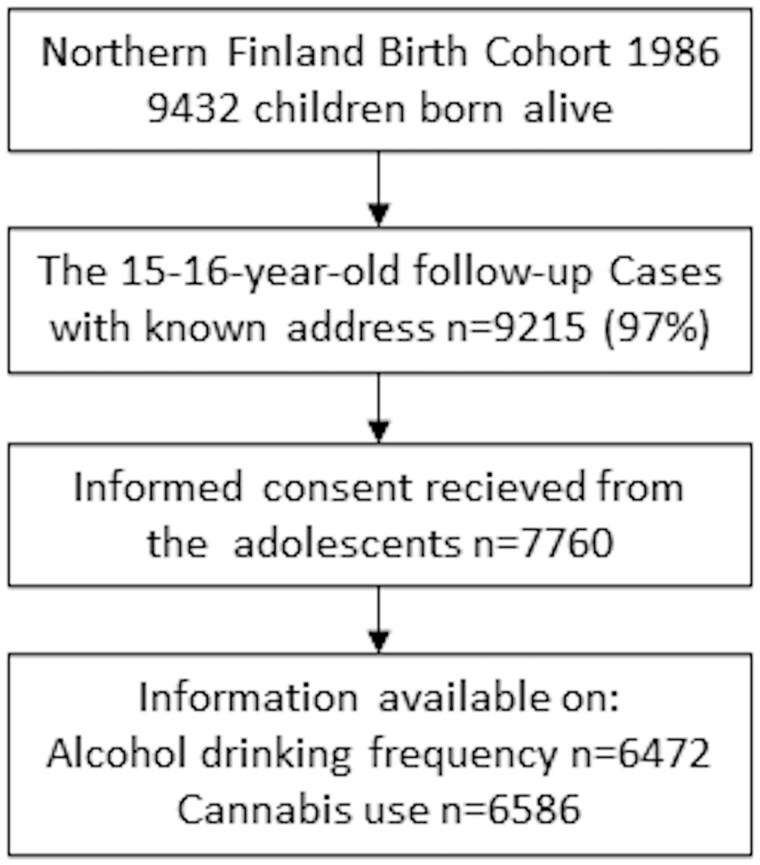
Flowchart of the study

The final sample included 7760 participants with information available on the frequency of alcohol use during the past 12 months (*n* = 6472) and on lifetime cannabis use (*n* = 6586) among the participants at ages 15–16 years (figure 1). Information on diagnoses were collected cumulatively from nationwide registers from the participants aged 16 years until 31 December 2018 (aged 32–33 years). The study was approved by the Ethics Committee of the Northern Ostrobothnia Hospital District in Finland on 15 January 2018 (EETTMK 108/2017).

### Exposures measured at ages 15–16 years

Frequency of alcohol intoxication was assessed with the question, ‘How many times have you been drunk during the past 12 months?’. Response options were (i) never, (ii) 1–2 times, (iii) 3–5 times, (iv) 6–9 times, (v) 10–19 times, (vi) 20–39 times, (vii) 40 times or more (University of Oulu^23^). This was categorized as a three-class variable: (i) never, (ii) 1–9 times and (iii) 10 times or more. This categorization was used to limit the group of frequently intoxicated adolescents to approximately 15% in line with previous studies of the same population.^18^^,^^21^

Lifetime cannabis use at ages 15–16 years was assessed with the question, ‘Have you ever tried or used marijuana or hashish?’ with these response options: ‘(i) never, (ii) once, (iii) 2–4 times, (iv) 5 times or more, (v) I use regularly’ (University of Oulu^23^). This was categorized as a two-class variable: (i) never and (ii) at least once. The categorization was used to provide a sufficient sample size.

### Outcomes

Data on diagnoses of TBI S06 (including S06.0–S06.9) and fractures of skull and facial bones S02 (including S02.0–S02.9), according to *International Classification of Disease*, Tenth Revision (ICD-10)^24^ until ages 32–33 years, were obtained from linkage to nationwide registers: The Care Register for Health Care 2001–18 of the National Institute for Health and Welfare and The Register of Primary Health Care Visits 2011–18. The Care Register contains information on patients discharged from inpatient care and all patient contacts in specialized outpatient care. The Register of Primary Health Care Visits includes all outpatient primary health care administered in Finland. The diagnosis was counted whether or not it was the primary diagnosis of the visit. Information on causes of death was obtained from the Population Register Data and Registry for Causes of Death covering all deaths in Finland.

### Potential confounders

Data on illicit substance use other than cannabis at ages 15–16 years were collected using a questionnaire during the field study. The participants were asked ‘Have you used ecstasy, heroin, cocaine, amphetamine, LSD or other similar intoxicating drugs?’. The use of inhalants was assessed by the question ‘Have you ever tried sniffing thinner, glue, etc. for intoxication?’. The misuse of medication was assessed by the two questions, ‘Have you ever tried or used medicines (sedatives, sleeping pills, or pain killers) for intoxication?’ and ‘Have you ever used alcohol and pills together?’. These data were pooled and participants were included in the ‘yes’ category if they answered ‘yes’ to any substance use-related question.

Adolescent mental health problems were assessed in the field study at ages 15–16 years using the Youth Self-report (YSR) questionnaire. YSR includes 112 items for rating 8 subscale symptoms. Part of them are referred to as externalizing problems (social problems and thought problems) (YSR-ext.) and part are referred to as internalizing problems (withdrawn, somatic complaints, anxiety and depression) (YSR-int.).^25^

Data on family background and parental education level were collected by a postal questionnaire to parents when the cohort members were aged 15–16 years. The family structure was classified as (i) both parents living with the subject all the time and (ii) all other families. Each parent’s education level was separately divided into two groups: (i) schooling for at least 12 years and (ii) schooling for <12 years.

### Statistical methods

All analyses were performed separately for males and females. The associations between head traumas and categorial variables describing substance use or background variables was studied with Pearson’s chi-square test or Fisher’s exact test; continuous variables were studied with Mann–Whitney *U* test. Those variables significantly associated with head traumas at univariate analyses were included in further models. Cox regression analysis with HRs and 95% CIs was used to study the association of lifetime cannabis use, frequency of alcohol intoxication and TBI. The reference group comprised abstinent adolescents who reported no experiences with alcohol or drugs. The probability of surviving without head trauma from ages 16 to 32–33 years in the study groups was determined with adjusted Cox regression survival analyses. The multivariable model included alcohol and cannabis use, family structure, mother’s education, previous head trauma (prior to age 16 years), use of any other drugs and YSR-externalizing symptoms score. Cox regression was used to test the interaction between variables. The statistical analyses were performed using SPSS statistical software (IBM SPSS Statistics, version 24; IBM Co., Armonk, New York, USA).

Attrition analyses regarding data collection at ages 15–16 years were presented by Miettunen et al.^26^ Fewer males than females participated (64% vs. 71%; χ^2^ test, *P* < 0.001). Participation was less common among adolescents with parental psychiatric disorder (58% vs. 69%, χ^2^ test, *P* < 0.001) and participants living in urban areas (66% vs. 71%, χ^2^ test, *P* < 0.001). The final outcomes were based on nationwide registers in which there were no missing data.

## Results

### Study population

Table 1 presents information separately for males and females on head trauma, self-reported alcohol intoxication frequency and cannabis use along with potential cofounders. In this cohort, 14% of males and 17% of females reported being intoxicated 10 times or more during the past year. Among males, 4% and among females, 5% had used cannabis at least once, respectively. By ages 32–33 years, *n* = 287 TBI and *n* = 325 craniofacial fractures had emerged. More than half of those diagnosed with TBI were male, as were 61% (*n* = 198/325) of those diagnosed with craniofacial fracture, respectively. A total of 2% of the study population had a diagnosis of head trauma prior to age 16 years.

**Table 1:** Sociodemographic characteristics and substance use at ages 15–16 and register-based TBI and craniofacial fracture diagnoses at the ages of 32–33 years in male and female

	No head trauma		All head trauma			TBI			Craniofacial fractures		
	Total *n* = 7189		Total *n* = 571			Total *n* = 287			Total *n* = 325		
	*n*	%	*N*	%	*P*-value	*n*	%	*P*-Value	*n*	%	*P*-Value
**Male**	3536	49	329	58	<0.001*	163	57	0.009*	198	61	<0.001*
**Family structure**											
Two parents	2347	78	198	73	0.051	96	70	0.027*	120	76	0.47
Other	654	22	73	27		41	30		39	25	
**Mother’s education**											
<12 years	2040	68	207	74	0.033*	105	75	0.091	129	78	0.009*
≥12 years	961	32	72	26		35	25		37	22	
**Head trauma prior to the age 16**											
No	3444	97	310	94	0.001*	154	95	0.051	187	94	0.002*
Yes	92	3	19	6		9	6		11	6	
**Frequency of alcohol intoxication during last 12 months**											
0	1173	41	67	25	<0.001*	32	24	<0.001*	40	25	<0.001*
1–9	1250	43	117	43		54	41		73	46	
≥10	468	16	83	31		47	35		45	29	
**Cannabis**											
No	2823	95	250	91	0.001*	120	87	<0.001*	152	94	0.54
Yes	140	5	26	9		18	13		10	6	
**Other drugs**											
No	2785	93	244	88	<0.001*	116	83	<0.001*	149	92	0.63
Yes	197	7	34	12		24	17		13	8	

**YSR-ext.** ^a^	**Mean/median**	**SD**	**Mean/median**	**SD**	***P*-value**	**Mean/median**	**SD**	***P*-value**	**Mean/median**	**SD**	***P*-value**
	
	12.4/11	7.6	14.4/13	8.3	<0.001*	15.5/13	9.3	<0.001*	13.8/13	7.5	0.021*

	***n***	**%**	***N***	**%**	***P*-value**	***n***	**%**	***P*-value**	***n***	**%**	***P*-value**

**Female**	3653	51	242	42	<0.001*	124	43	0.009*	127	39	<0.001*
**Family structure**											
Two parents	2407	77	139	68	0.003*	64	66	0.011*	78	68	0.033*
Other	708	23	65	32		33	34		36	32	
**Mother’s education**											
<12 years	2044	66	142	72	0.12	64	68	0.77	83	75	0.065
≥12 years	1037	34	56	28		30	32		28	25	
**Head trauma prior to the age 16**											
No	3585	98	233	96	0.044*	117	94	0.011*	124	98	0.74
Yes	68	2	9	4		7	6		3	2	
**Frequency of alcohol intoxication during last 12 months**											
0	1104	35	62	30	0.10	26	25	0.012*	38	34	0.95
1–9	1404	45	95	46		47	45		50	45	
≥10	598	19	51	25		31	30		23	21	
**Cannabis**											
No	2952	94	184	87	<0.001*	85	82	<0.001*	106	93	0.93
Yes	184	6	27	13		19	18		8	7	
**Other drugs**											
No	2714	86	169	80	0.011*	75	71	<0.001*	99	87	0.71
Yes	441	14	43	20		30	29		15	13	

**YSR-ext.** ^a^	**Mean/median**	**SD**	**Mean/median**	**SD**	***P*-value**	**Mean/median**	**SD**	***P*-value**	**Mean/median**	**SD**	***P*-value**

	15.5/14	8.1	16.6/16	9.2	0.08	17.6/17	9.6	0.016*	15.4/15	8.4	0.94

SD, standard deviation.

a Information of YSR externalizing problems are reported as continuous variables.

* *P*-values <0.05, categorical variables tested with χ^2^ test or Fischer’s exact test and continuous variables with Mann–Whitney *U* test.

A total of 1% (*n* = 77) of the participants were lost from follow-up due to death. Head trauma was not reported as the primary cause of death for any of the cohort members, even though a majority (73% *n* = 56) of the deaths were classified as accidents or suicides. (Data available from authors on request.)

### Associations

Sex was statistically significantly associated with both TBI and craniofacial fractures in the bivariate examination. Alcohol intoxication frequency was associated with TBI in both males and females and with craniofacial fractures in males. Furthermore, using cannabis at least once was associated with TBI in males and females, but no statistically significant difference between the groups was seen with craniofacial fractures in either males or females. Use of any drugs other than cannabis was associated significantly with TBI in both males and females (table 1).

Sex and frequent (10 times or more) alcohol intoxication associated with all outcomes in univariate Cox regression. All the variables except parental education level are associated with TBI. Previous head trauma and mother’s education level associated with craniofacial fractures. Father’s education level did not associate with any of the outcomes (Supplementary table S1).

In interaction analysis, a significant interaction existed between alcohol intoxication frequency (as a two-class variable 0–9 times/10 times or more) and sex (HR 0.6, 95% CI 0.4–0.9, *P* = 0.018), indicating that the effect of frequent alcohol intoxication in adolescence on elevation of the risk for head trauma is lower for females than for males.

The multivariate model was adjusted for the following variables: family structure, mother’s education, information on previous head trauma, YSR-externalizing symptoms, alcohol intoxication frequency, cannabis use and other substance use at ages 15–16 years. Frequent alcohol intoxication was not statistically significantly associated with the risk of injuries among females in this model, but the association among males remained statistically significant for both TBI (HR 2.6, 95% CI 1.7–3.9, *P* < 0.001) and craniofacial fractures (HR 2.7, 95% CI 1.6–4.8, *P* < 0.001). In turn, cannabis use remained statistically significantly associated with a greater risk of TBI among females (HR 1.9, 95% CI 1.1–3.2, *P* = 0.024), while the association in males was not statistically significant. Misuse of any other drugs but cannabis is also associated with the risk of any head trauma in females (table 2).

**Table 2:** Adolescent alcohol and cannabis use predict head traumas in later life in male and female respectively

	All head trauma			TBI			Craniofacial fractures		
	HR	CI 95%	*P*-value	HR	CI 95%	*P*-value	HR	CI 95%	*P*-value
**Male**									
**Frequency of alcohol drinking during last 12 months**									
1–9 vs. 0	1.2	0.7–1.9	0.57	1.5	1.1–2.2	0.02*	2.0	1.3–3.1	0.004*
≥10 vs. 0	2.4	1.3–4.1	0.003*	2.6	1.7–3.9	<0.001*	2.7	1.6–4.8	<0.001*
**Cannabis**									
Yes vs. no	1.1	0.6–2.2	0.73	1.2	0.7–2.0	0.58	1.1	0.5–2.3	0.89
**Other drugs**									
Yes vs. no	1.7	1.0–3.0	0.072	1.2	0.7–1.9	0.54	0.5	0.2–1.3	0.15
**Family structure**									
Other vs. two parents	1.3	0.9–2.1	0.19	1.1	0.8–1.6	0.52	0.9	0.6–1.4	0.66
**Mother’s education**.									
≥12 vs. <12 years	0.7	0.4–1.1	0.13	0.7	0.5–1.0	0.058	0.6	0.4–1.0	0.035*
**Head trauma prior to the age 16**									
Yes vs. no	1.6	0.6–3.9	0.33	1.6	0.8–3.2	0.17	1.6	0.7–4.0	0.30
**YSR**–**ext.**^b^	1.03	1.03–1.05	0.03*	1.02	1.00–1.03	0.11	1.01	1.0–1.04	0.46
**Female**									
**Frequency of alcohol drinking during last 12 months**									
1–9	0.9	0.5–1.7	0.84	1.0	0.7–1.5	0.98	1.1	0.7–1.8	0.72
≥10	1.3	0.7–2.7	0.42	1.1	0.7–1.8	0.57	1.1	0.6–2.1	0.70
**Cannabis**									
Yes vs. no	2.2	1.1–4.4	0.023*	1.9	1.1–3.2	0.024*	1.1	0.45–2.77	0.81
**Other drugs**									
Yes vs. no	2.1	1.1–3.7	0.018*	1.3	0.8–2.1	0.23	0.9	0.4–1.7	0.68
**Family structure**									
Other vs. two parents	1.2	0.7–2.0	0.53	1.3	0.9–1.8	0.21	1.4	0.9–2.2	0.16
**Mother’s education**									
≥12 vs. <12 years	0.9	0.6–1.5	0.78	0.8	0.6–1.2	0.26	0.8	0.5–1.2	0.25
**Head trauma prior to the age 16**									
Yes vs. no	4.8	2.1–11.2	<0.001*	2.5	1.2–5.4	0.017*	1.1	0.3–4.6	0.86
**YSR-ext.**^a^	1.00	0.97–1.03	0.73	1.00	0.98–1.02	0.67	0.99	0.96–1.02	0.50

Notes: Multivariate model adjusted for alcohol intoxication frequency, cannabis use, use of other drugs, family structure, mother’s education, previous head trauma and YSR-externalizing problems.

a Information for YSR externalizing problems are reported as continuous variables.

* *P*-values <0.05, Cox regression.

**Figure 2:**
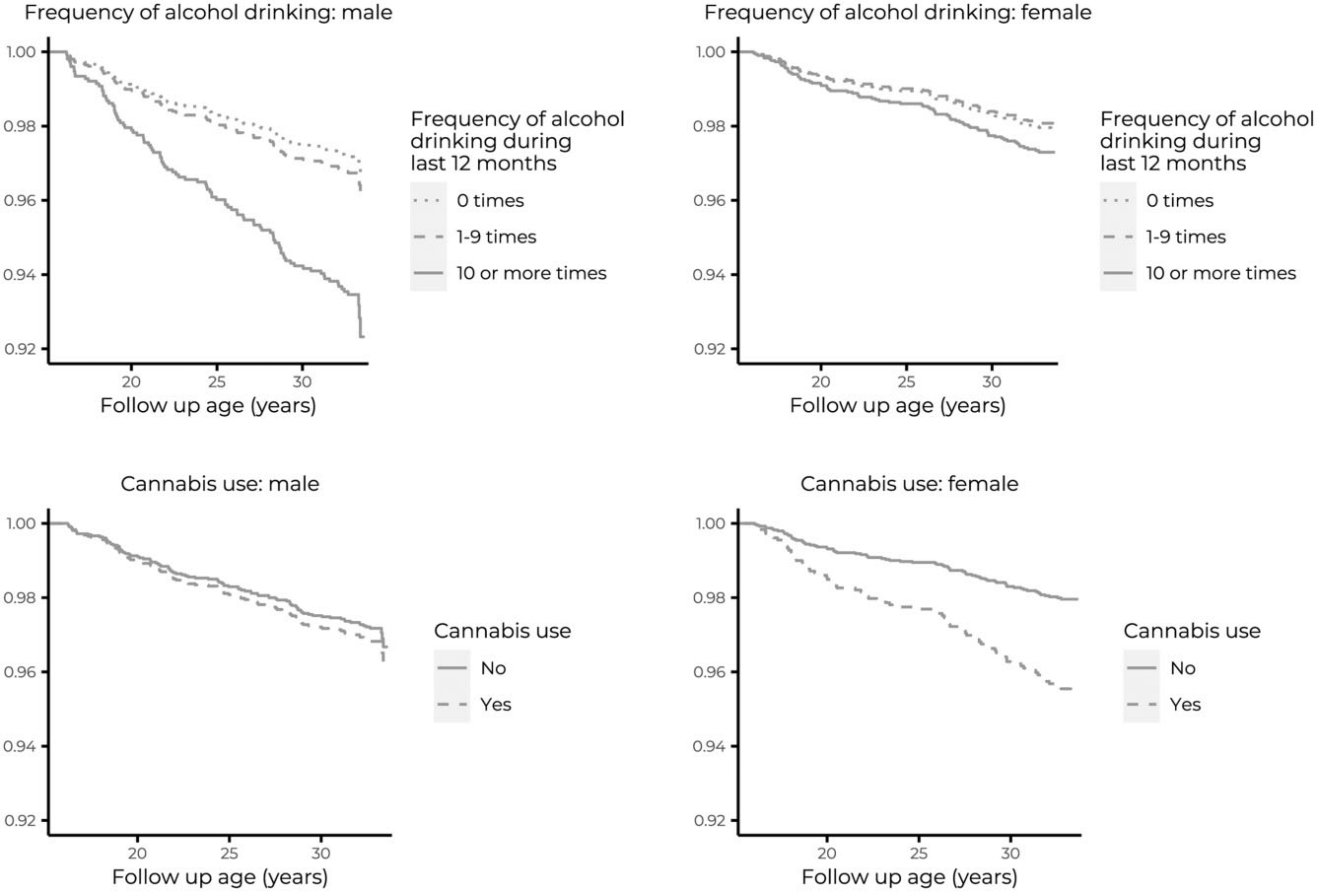
TBI (including ICD-10 codes S06.0–S06.9) free survival as functions of adolescent alcohol intoxication frequency and cannabis use for males and females

Figure 2 presents the survival curves for TBI, alcohol intoxication frequency and cannabis use separately for males and females.

## Discussion

In this large birth cohort study, we have studied the relationship between alcohol intoxication frequency and cannabis use at ages 15–16 years and the risk of subsequent head trauma requiring medical attention by ages 32–33 years. Here, adolescent cannabis use was associated with an elevated risk of TBI for females, and this association remained statistically significant after adjustments for family structure, mother’s education, use of any other drugs, previous head trauma and YSR-externalizing symptoms score. This association was not statistically significant for males. Furthermore, frequent alcohol intoxication in adolescence is associated with an increased risk of both TBI and craniofacial fractures for males but not for females in our study. For males, this is in line with previous findings indicating that frequent alcohol intoxication increases the risk for both TBI and craniofacial fractures.^1^^,^^5^^,^^6^^,^^15^ In the study by Winqvist et al.^6^ alcohol drinking was particularly associated with head injuries among males. They did not report a similar association among females, but in their cohort alcohol drinking was also much scarcer among females while in our cohort the proportion of frequent drinkers was roughly equal in both groups.

Cannabis use in adolescence was an independent risk factor for subsequent TBI for females in our study. To our knowledge, there are no previous longitudinal studies that have examined adolescent cannabis use as an independent risk factor for TBI. Eskander et al.^5^ found no statistically significant association in their nationwide cross-sectional study between cannabis use disorder and hospitalization due to TBI in childhood and adolescence (ages 8–18 years). In their study, AUD was associated with increased risk for hospitalization, and cannabis use was more prevalent among those adolescents with AUD.^5^ Cannabis use has been associated with a range of harms including health problems and societal problems, that also may associate with elevated risk of head traumas.^27^ Cannabis use has been recognized as a risk factor for traffic accidents^17^ and impaired driving^18^ in the general population. Motor vehicle collisions are among the most common causes of TBI hospitalization in Europe.^1^^,^^2^

Frequent alcohol intoxication in adolescence was an independent risk factor for subsequent TBI and craniofacial fractures in males that is in line with previous findings. Emerging evidence exists that males and females vary significantly in the incidence and consequence of TBI, especially in adolescence and young adulthood. Males have a higher incidence of TBI, especially in adolescence and young adulthood, and are more often intoxicated at the time of injury. The variation is partly explained by differences in pre-injury substance use patterns. Binge drinking prior to TBI has been reported to be more prominent in males.^28^

Our study also has certain limitations. The data in national registers are generally reliable, but the accuracy may vary, for example, under-recording of subsidiary diagnoses is a known limitation for register data.^29^ There is information neither on severity of the head trauma nor on acute intoxication at the time of injury. Also, as the information on adolescent substance use was collected in Northern Finland during the years 2000–2001, it may have an influence on generalization of our results, particularly in more urban areas. The information on alcohol and cannabis use in adolescence is based on self-reported data, and no objective measurements of blood alcohol or cannabinoid levels were done. Other studies have commented favorably on the reliability of adolescent self-reported alcohol use.^30^ The information on the frequency of intoxication was retrospectively estimated by the participants. Lastly, there is no information available on continuing the alcohol and cannabis use in adulthood. Self-reported cannabis use was relatively rare in this cohort compared with a national survey on drug use.^11^ In addition, to ensure adequate statistical power in our multivariable analysis, we employed a dichotomized variable for cannabis use, which may undermine the association between TBI and cannabis use. Combined with the lesser participation of males,^26^ the lacking significance of the association between cannabis use and TBI in males may be biased.

The strengths of the present study are its longitudinal prospective design with a considerable follow-up time, its large sample size in a general population cohort and the combined data from nationwide registers. The models in this study were adjusted for a range of confounders, and multiple substance use markers were studied simultaneously.

## Conclusions

This is the first prospective study to report associations between adolescent cannabis use and an elevated risk of TBI, a finding to be replicated in future studies. Our findings strengthen the previous findings indicating that frequent alcohol intoxication in adolescence associates with a higher risk of head traumas in later life. Our results also suggest that a variation in risk factors exists between males and females. Further investigation is needed to find the possible explanations behind this variation. The possibility of separate risk factors for males and females should be regarded in future studies on TBI risk factors. Detection of and interventions in adolescent frequent alcohol use and cannabis experiments are encouraged. Awareness of the risk factors for head traumas should be increased.

## Supplementary Material

ckad151_Supplementary_Data

## Data Availability

The data underlying this article were accessed from Northern Finland Birth Cohort 1986 (https://www.oulu.fi/en/university/faculties-and-units/faculty-medicine/northern-finland-birth-cohorts-and-arctic-biobank/nfbc-aineistopyynto), The Care Register for Health Care of the National Institute for Health and Welfare and The Register of Primary Health Care (https://findata.fi/en/data/). The derived data generated in this research will be shared on reasonable request to the corresponding author. Key points Adolescent cannabis use was associated with an elevated risk of traumatic brain injury for females, and this association remained statistically significant after adjustments for various cofounders. Frequent alcohol intoxication in adolescence is associated with an increased risk of both traumatic brain injury and craniofacial fractures for males. Further investigation is needed to find the possible explanations behind the variation between males and females concerning head injury risk factors. The possibility of separate risk factors for males and females should be regarded in future studies on risk factors of traumatic brain injuries. Awareness of the risk factors for head traumas should be increased among the public.
