# Supplementary material for: Adolescent alcohol and cannabis use as risk factors for head trauma in the Northern Finland Birth Cohort study 1986
Source: Eur J Public Health. 2023 Aug 23;33(6):1115–21. doi: 10.1093/eurpub/ckad151 (PMC10710361; doi:10.1093/eurpub/ckad151)
Supplement: ckad151_Supplementary_Data [file ckad151_supplementary_data.pdf]

SupplementaryTable 1. Prediction of head trauma by substance use and other variables at the age of 15-16 years.

	All head trauma			TBI[1]			Craniofacial fractures		
	**HR**	**CI 95%**	**p-value**	**HR**	**CI 95%**	**p-value**	**HR**	**CI 95%**	**p-value**
**Sex**	0.7	0.6-0.9	<0.001*	0.8	0.6-1.0	0.022*	0.6	0.5-0.8	<0.001*
female vs. male									
**Frequency of alcohol intoxication during last 12 months**									
1-9 vs. 0	1.4	1.1-1.7	0.002*	1.3	1.0-1.7	0.092	1.5	1.1-1.9	0.004*
10 or more vs. 0	1.8	1.4-2.4	<0.001*	2.2	1.6-3.2	<0.001*	1.7	1.1-2.4	0.008*
**Cannabis**	2.1	1.6-2.8	<0.001*	3.0	2.1-4.2	<0.001*	1.2	0.7-1.9	0.53
yes vs. no									
**Other drugs**	1.6	1.3-2.1	<0.001*	2.4	1.8-3.3	<0.001*	0.9	0.6-1.4	0.81
yes vs. no									
**Family structure**	1.4	1.2-1.7	0.001*	1.6	1.2-2.1	0.001*	1.3	1.0-1.7	0.066
other vs. two parents									
**Mother's education**	0.8	0.6-0.9	0.008*	0.8	0.6-1.1	0.14	0.6	0.5-0.8	0.001*
12 yrs. or more vs less than 12 yrs.									
**Father's education**	1.0	0.8-1.3	1.0	1.1	0.8-1.5	0.67	0.9	0.6-1.2	0.45
12 yrs. or more vs less than 12 yrs.									
**Head trauma prior to the age 16**	2.3	1.3-3.1	<0.001*	2.5	1.5-4.1	<0.001*	1.9	1.1-3.2	0.019*
yes vs. no									
**YSR-ext.**[2]	1.02	1.01-1.03	<0.001*	1.03	1.02-1.05	<0.001*	1.01	0.99-1.02	0.46

*p-values <0.05, Cox regression

[1]Traumatic brain injury

[2]Information for Youth Self Report (YSR) externalizing problems are reported as continuous variables.
